# Geometrical Selection of GaN Nanowires Grown by Plasma-Assisted MBE on Polycrystalline ZrN Layers

**DOI:** 10.3390/nano13182587

**Published:** 2023-09-19

**Authors:** Karol Olszewski, Marta Sobanska, Vladimir G. Dubrovskii, Egor D. Leshchenko, Aleksandra Wierzbicka, Zbigniew R. Zytkiewicz

**Affiliations:** 1 Institute of Physics, Polish Academy of Sciences, Al. Lotnikow 32/46, 02-668 Warsaw, Polandzytkie@ifpan.edu.pl (Z.R.Z.); 2 Faculty of Physics, St. Petersburg State University, Universitetskaya Embankment 13V, 199034 St. Petersburg, Russia; dubrovskii.ioffe@mail.ru (V.G.D.);

**Keywords:** GaN nanowires, ZrN buffer layers, molecular beam epitaxy, nanowire orientation, geometrical selection

## Abstract

GaN nanowires grown on metal substrates have attracted increasing interest for a wide range of applications. Herein, we report GaN nanowires grown by plasma-assisted molecular beam epitaxy on thin polycrystalline ZrN buffer layers, sputtered onto Si(111) substrates. The nanowire orientation was studied by X-ray diffraction and scanning electron microscopy, and then described within a model as a function of the Ga beam angle, nanowire tilt angle, and substrate rotation. We show that vertically aligned nanowires grow faster than inclined nanowires, which leads to an interesting effect of geometrical selection of the nanowire orientation in the directional molecular beam epitaxy technique. After a given growth time, this effect depends on the nanowire surface density. At low density, the nanowires continue to grow with random orientations as nucleated. At high density, the effect of preferential growth induced by the unidirectional supply of the material in MBE starts to dominate. Faster growing nanowires with smaller tilt angles shadow more inclined nanowires that grow slower. This helps to obtain more regular ensembles of vertically oriented GaN nanowires despite their random position induced by the metallic grains at nucleation. The obtained dense ensembles of vertically aligned GaN nanowires on ZrN/Si(111) surfaces are highly relevant for device applications. Importantly, our results are not specific for GaN nanowires on ZrN buffers, and should be relevant for any nanowires that are epitaxially linked to the randomly oriented surface grains in the directional molecular beam epitaxy.

## 1. Introduction

III-nitride nanowires (NWs) provide an attractive alternative for planar structures in micro- and optoelectronic devices. Due to a small footprint in contact with the substrate surface, NWs are free of misfit dislocations, even when grown on highly lattice-mismatched substrates [1]. As a result, NWs are of a superior structural quality that is not achievable in comparable planar heterostructures [2,3,4]. The process of self-assembled growth of GaN NWs by molecular beam epitaxy (MBE) has been thoroughly investigated, especially for amorphous substrates such as nitridated Si [1,4,5,6,7,8,9], SiO_x_ [10,11] and Al_x_O_y_ [12,13,14]. A specifically attractive benefit offered by the self-assembled growth of GaN NWs on such substrates is that the epitaxial constraints are very weak and the NWs grow perpendicular to the substrate surface [9]. Consequently, ensembles of well-aligned vertical NWs of equal height are formed, which is crucial for the subsequent processing and fabrication of NW-based light emitting devices such as LEDs. Unfortunately, SiO_2_, Al_x_O_y_ and SiN_x_ buffer layers form non-ohmic electrical contacts to GaN. This hinders carrier transport and heat dissipation at the NW/substrate interface [15]. Furthermore, due to the optical transparency of SiO_2_, Al_x_O_y_ and SiN_x_ buffers, a large part of light generated in the LED quantum well is lost by its absorption in a Si carrier wafer. Thus, various alternative substrates for GaN NW-based devices are still being tested. 

There is increasing interest in growing GaN NWs on metal substrates [16,17,18,19,20,21,22,23,24]. Such substrates exhibit excellent electrical and thermal conductivities as well as a high optical reflectance. However, direct MBE growth of GaN NWs on metallic surfaces is challenging. Calabrese et al. [22] reported efficient reactions of TiN/sapphire substrates with the impinging Ga flux, resulting in the surface roughening at high temperatures. Macroscopic delamination of Mo and Ti films from a Si substrate at a high temperature was also observed [16]. Since GaN NWs are epitaxially linked to the underlying metal [17,20], spatial orientation of the NWs is determined by the mosaic structure of the metallic surface. For example, May et al. [18] observed GaN NWs uniformly tilted with respect to the surface normal within the individual grains of polycrystalline Ta and Ti foils. Transmission electron microscopy studies of GaN NWs on TiN substrates clearly showed the epitaxial relationships [2 − 1 − 10] GaN//[011]TiN and (0002)GaN//(111)TiN [20]. Thus, GaN NWs are usually locally textured with respect to the underlining metallic grains, eventually making them impractical for applications requiring vertical NW ensembles. The growth of GaN NWs on monocrystalline metallic substrates of proper crystallographic orientation solves the problem [24], but makes the whole fabrication procedure much more complicated. Interestingly, the high stability of metallic TiN buffers allowed the recent successful PAMBE growth of high-quality AlN NWs at an exceptionally high growth temperatures of ~1200 °C [25,26].

In this paper, we report plasma-assisted MBE (PAMBE) growth of GaN NWs on thin polycrystalline ZrN buffer layers, sputtered onto Si(111) substrates. We experimentally study and describe, within a dedicated model, the growth rate of GaN NWs as a function of the Ga beam angle and the NW tilt angle (induced by the randomly oriented metallic grains), with and without substrate rotation. It is demonstrated that the MBE system geometry has the direct and dominant influence on the NWs’ orientation. The axial NW growth rate generally decreases with the NW tilt angle, which is why vertically aligned NWs grow faster than inclined NWs and become dominant in the ensemble for longer growth times or higher NW surface densities. It is shown that uniform ensembles of vertical GaN NWs, as required in device applications, can be obtained on polycrystalline ZrN buffer at a high enough NW density, despite the random orientation of metallic grains on which the epitaxially linked NWs nucleate. The geometry of the MBE overcompensates for the random orientation of initial GaN crystallites induced by metallic grains. Importantly, these results, including the effect of geometrical selection of vertically aligned NWs, are not limited to a particular material system, but rather are inherent to NW growth via the directional MBE technique in general.

## 2. Materials and Methods

The NW samples were grown by PAMBE in a RIBER Compact 21 system, using an Addon radio frequency nitrogen source and a solid-source effusion Ga cell. Si(111) substrates were covered with a 20 nm thick ZrN buffer layer using DC sputtering from a ZrN target. Layer deposition was performed at room temperature under an Ar pressure of 1 × 10^−2^ mbar and a deposition rate of 0.3 nm/s. The ZrN/Si substrates were transferred in air to the PAMBE system, heated to 150 °C for 1h in the loading chamber, and subsequently to 500 °C for 5h in the preparation chamber to remove any volatile contaminating species before the transfer to the growth chamber. After heating the substrate to a required temperature, the PAMBE growth process was initiated by simultaneously opening the Ga and N shutters. GaN NWs were grown under N-rich conditions keeping N and Ga impinging fluxes of Ф_N_ = 16 nm/min and Ф_Ga_ = 8 nm/min, respectively. Cross-sectional scanning electron microscopy (SEM) of thick GaN(0001) films grown under slightly N- and Ga-rich conditions at low temperatures (680 °C) was used to calibrate the Ga and N fluxes in the 2D-equivalent growth rate units of nm/min [27]. The stability of the N flux was controlled during growth using an optical sensor of plasma light emission attached to the plasma cell [28]. In the standard growth experiments, the substrate was rotated at 6 rpm. However, experiments without substrate rotation were performed as well. The morphology of as-grown GaN NW ensembles was examined using a field-emission Hitachi Su-70 SEM (Tokyo, Japan). X-ray diffraction (XRD) measurements of the ZrN/Si substrates as well as of GaN NWs on ZrN buffers were performed using a Panalytical X’Pert Pro-MRD diffractometer (Malvern, UK) equipped with an X-ray lamp with Cu anode, hybrid two-bounce Ge (220) monochromator and Soller slits in front of a Pixcel detector. 

## 3. Results and Discussion

### 3.1. Microstructure of ZrN Buffer Layers on Si(111) Substrates

**Figure 1:**
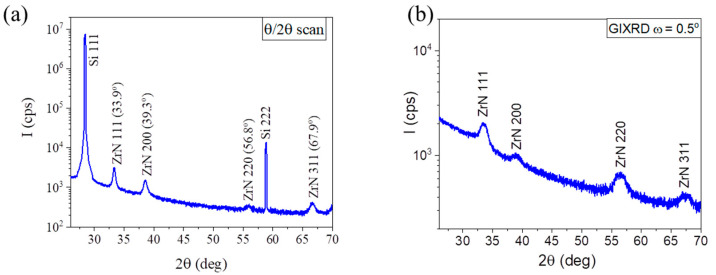
(**a**) θ/2θ X-ray diffraction scan and (**b**) GIXRD scan of ZrN polycrystalline film on Si(111) substrate.

Figure 1a shows the θ/2θ scans of ZrN layer on Si(111) substrate, measured in a wide range of diffracted angles. The XRD signals from Si(111) substrate and from polycrystalline cubic ZrN layer (indicated via JCPDF card No. 00-031-1493) are clearly visible. To eliminate the risk that another lines, originating, for example, from orthorhombic Zr_3_N_4_ phase, are overlapped by strong Si peaks, we also measured the same sample by grazing incidence X-ray diffraction (GIXRD) [29] using a beam incident angle of ω = 0.5°. As seen in Figure 1b, the diffracted signal from Si(111) substrate was eliminated, while the ZrN lines were identical to those observed under the standard XRD geometry. This proves the single-phase polycrystalline cubic structure of the ZrN film. Importantly, we observed that the angular positions of XRD ZrN lines did not change after GaN NW growth. In order to determine the average grain size of ZrN, we used the size–strain plot procedure [30]. The grain size was estimated by fitting the 111, 200, 220 and 311 diffraction peaks using the Voight function. The Lorentzian component of the full width at half maximum (FWHM) was then used for determining the average crystalline size with different orientation using the Debye–Scherrer formula [31]. Such a procedure resulted in a grain size in our ZrN films of about 20 nm. In addition, atomic force microscopy studies evidenced the very smooth surfaces of the buffer layers with an RMS value of 0.15 nm.

### 3.2. Arrangement of GaN NWs on ZrN/Si Substrates

**Figure 2:**
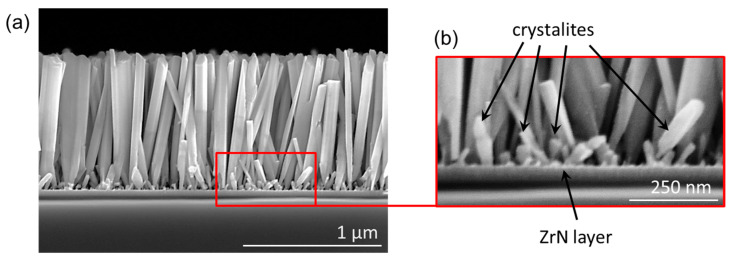
(**a**) SEM image of GaN NWs grown on ZrN buffer. The NWs are mostly oriented along the substrate normal. (**b**) Small GaN crystallites between the NWs.

Figure 2a shows an SEM image of a typical GaN NW ensemble obtained on polycrystalline ZrN buffer. The NWs have an average diameter of 70 nm and average length of around 1 μm. The NW density determined from plane view SEM images is ~1 × 10^11^ cm^−2^, which is a typical value for self-assembled GaN NWs on nitridated Si substrates [10,11] or Al_x_O_y_ [32] buffers_._ The NWs are mostly aligned along the substrate normal. This is a surprising finding, because the initial orientation of the NWs should be directly determined by the formation of epitaxial link between the NWs and the randomly oriented substrate grains. Additionally, small GaN crystallites marked by arrows in Figure 2b are clearly seen on the substrate surface between the NWs.

**Figure 3:**
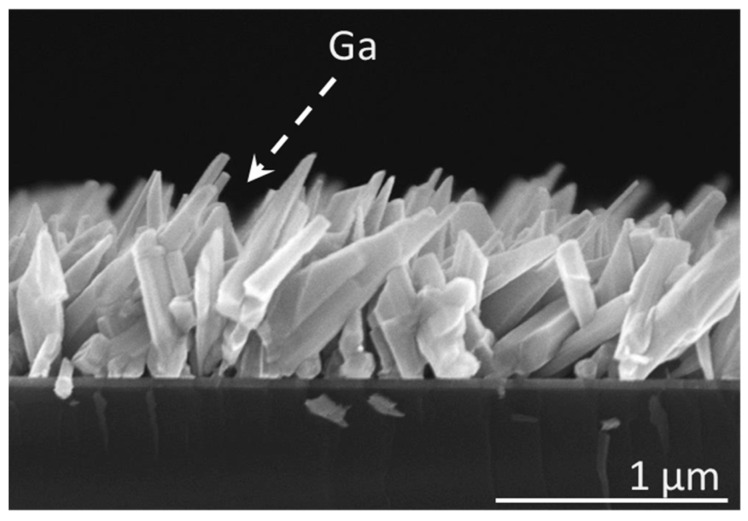
SEM image of GaN NWs grown on ZrN buffer without substrate rotation. The sample was cleaved in the plane defined by the normal to the substrate and the line-of-sight of the Ga source. The dashed line shows the Ga beam direction in the MBE system.

Quite different growth behavior was observed when the NWs were formed under the same growth conditions but without substrate rotation. Figure 3 shows the SEM image of GaN NW ensemble formed in that way. The surface density of these NWs is similar to that obtained with substrate rotation. However, instead of adopting vertical orientation, most NWs grew inclined in a specific direction. The sample was cleaved in the plane defined by the substrate normal and the line-of-sight of the Ga source. During the SEM measurements, the sample position was carefully adjusted to keep the sample cross-section parallel to the imaging plane. Therefore, we can conclude from Figure 3 that inclined NWs point in the Ga beam direction, which makes an angle of 40° to the substrate normal in our MBE system. Such behavior is similar to that reported by Foxon et al. [33], and indicates a dominant impact of the MBE chamber geometry on the NW orientation.

**Figure 4:**
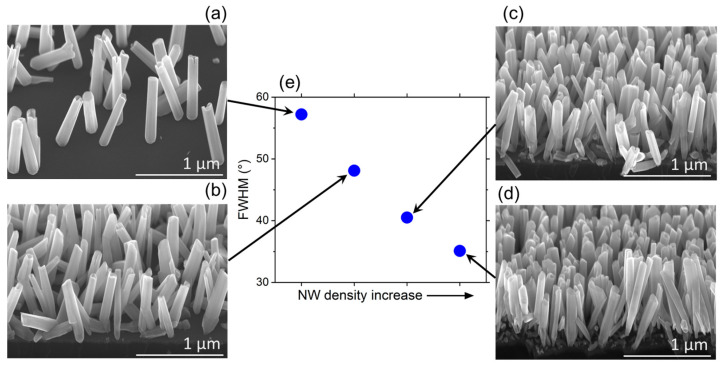
(**a**–**d**) SEM images of different parts of GaN NW sample with increasing NW density, (**e**) shows the FWHM values of the XRD ω-scan representing the tilt dispersion of the NW ensemble at the respective parts of the sample.

In additional experiment, we grew GaN NWs on ZrN/Si substrate mounted in the holder that induced a temperature gradient, and thus a substantial NW surface density gradient, across the substrate. According to Figure 4, the NWs’ orientation strongly depends on their density. For the sparse ensemble (Figure 4a), the NW orientation appears random, following the epitaxial link to the randomly oriented ZrN grains. The previously observed small crystallites between the NWs are absent. SEM images in Figure 4b–d show that the NW tilt dispersion significantly decreases for higher NW density. To quantify these observations, the XRD technique was used to measure the tilt angle of GaN NWs on ZrN buffer layer with respect to the substrate normal. In our XRD experiments, the X-ray spot size in the substrate plane was reduced to <2 mm. This enables local measurements of the FWHM of X-ray ω scan for NW ensembles with different surface densities. Figure 4e shows the FWHM of XRD ω scans from different parts of the sample. The plot clearly indicates that the FWHM value strongly decreases with the NW density, meaning that the dispersion of the NW orientations is reduced in denser NW ensembles. In the part with sparse NWs (Figure 4a), the NW tilt spread equals ±30°. This indicates nearly random orientation of the NWs as determined by the random orientation of ZrN grains in the polycrystalline buffer layer. Conversely, in the part with dense NWs (Figure 4d), the tilt dispersion decreases to ±18°. This value is still much larger than for self-assembled GaN NWs on nitridated Si substrates [34] or on amorphous Al_x_O_y_ buffers [32]. It should be noted, however, that small crystallites that are present between dense GaN NWs (see Figure 2b) are randomly oriented and contribute to the broadening of the X-ray diffraction curve. This leads to overestimation of the FWHM value and therefore the NW tilt dispersion. Therefore, in agreement with the results of SEM studies (Figure 4d), the real tilt dispersion in the upper sections of the densely packed NW ensembles is significantly lower than the value of ±18° found from the XRD measurements. Such well-oriented NW ensembles are well suitable for fabrication of devices such as LEDs or detectors, where uniform alignment of the NWs is essential.

The experimental results presented above indicate that a specific mechanism must exist which determines a percentage of vertically aligned NWs in the ensemble, depending on the MBE chamber geometry and the NW surface density. The MBE growth scenario includes a competition between the epitaxial constraints for NWs nucleating with random orientations on the metallic grains, different growth rates of NWs having different tilt angles and being influenced by substrate rotation, and the shadowing effect, which suppresses the MBE growth of shorter NWs. In the following section, we present a general model that predicts the NW axial growth rate as a function of the NW tilt angle, group III (Ga) beam angle, and includes the substrate rotation in the directional MBE technique. 

### 3.3. Modelling MBE Growth Rates of Inclined NWs

The modeling of the axial growth rate of the inclined catalyst-free GaN NWs in the directional MBE technique with substrate rotation is based on the following assumptions. First, we consider Ga-limited growth under N-rich conditions, which are typical for PAMBE of III-nitride NWs [6,11,12,14,35,36,37,38,39,40,41], and definitely holds in our experiments. Second, we consider the contributions originating from Ga flux impinging onto the top facet of cylindrical (or hexahedral) NW of radius R and the upper part of its sidewalls of length $\lambda_{SW}$, which equals the diffusion length of Ga adatoms on the NW sidewalls [36,37,38,39,40]. The contribution from Ga adatoms diffusing from a substrate surface, which is usually included in the growth modeling of GaAs and other III-V NWs [42,43], is neglected due to a short diffusion length of Ga adatoms at high temperatures employed for GaN NW growth. The estimates of Refs. [36,37,40] yield $\lambda_{SW}≅40\pm 5$ nm in a temperature range from 760 to 800 °C. Therefore, diffusion from a substrate surface becomes ineffective for GaN NWs longer than ~100 nm. Third, for a rotating substrate, we use the growth rate averaged over one substrate rotation, as in Ref. [43], for inclined Au-catalyzed GaAs NWs grown by MBE. Fourth, we consider the MBE growth of individual NWs without the shadowing effect, which will be introduced later. 

**Figure 5:**
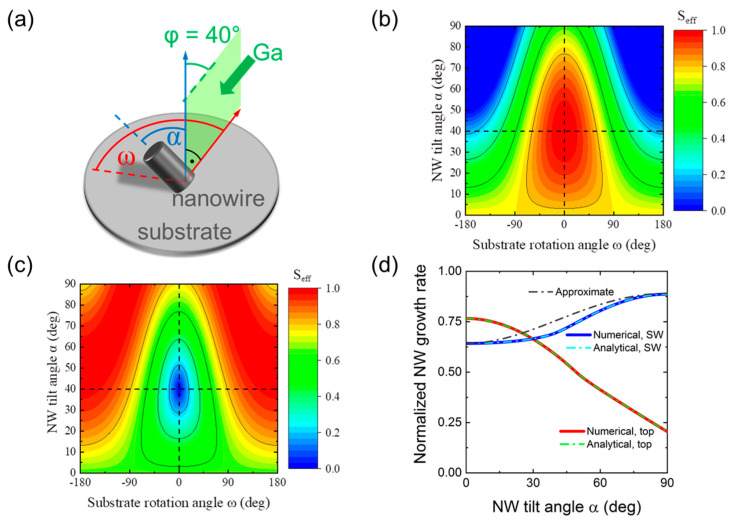
(**a**) Illustration of MBE growth of an inclined NW with the tilt angle α, from a Ga beam with the angle φ= 40°; ω denotes the angle related to substrate rotation. Effective area $S_{eff}$ of (**b**) the NW top facet and (**c**) NW sidewalls as a function of α and ω at a fixed φ= 40°. Horizontal dashed lines in (**b**,**c**) mark the Ga supply direction. (**d**) Normalized contributions of the top and sidewall (SW) facets into the total axial growth rate versus the NW tilt angle at φ= 40°. Numerical solutions are obtained by summing up the elements of the contour plots of (**b**,**c**) over ω at a fixed α and dividing the result by the number of elements. Analytical solutions are obtained from Equations (2)–(4). The approximate solution is obtained using Equation (5).

Figure 5a shows the geometry of a catalyst-free GaN NW with flat top, inclined by the angle α to the substrate normal and subjected to Ga beam inclined by the angle φ to the substrate normal, with ω as the third angle related to substrate rotation (0≤ω<2π). From geometrical considerations, the angle ξ between the Ga beam and the NW growth axis is given by cosξ=sin φsinαsinω+cos φcosα. When cosξ<0, no Ga flux impinges onto the top NW facet. For the axial NW growth rate averaged over one substrate rotation, we use

(1) $$\frac{1}{v}\frac{dL}{dt}=\langle \cos\xi \rangle +\langle \sin\xi \rangle \frac{2\lambda_{SW}}{\pi R}$$

with v as the 2D equivalent deposition rate of Ga atoms. The values of cosξ and sinξ are averaged over ω:

(2) $$\langle \cos\xi \rangle =\frac{1}{2\pi }\int_{0}^{2\pi }\cos\xi d\omega ,\;\;\;\;\;\;\langle \sin\xi \rangle =\frac{1}{2\pi }\int_{0}^{2\pi }\sin\xi d\omega .$$

Here,

(3) $$\;\;\;\;\;\;\;\;\;\;\;\;\;\;\;\;\;\;\;\;\;\;\;\;\;\cos\xi =\{\begin{array}{c} sin\;\varphi sin\alpha sin\omega +\cos\;\varphi \cos\alpha ,\;\cos\xi \geq 0\\ 0,\;\;\;\cos\xi <0 \end{array}$$

and

(4) $$\sin\xi =\sqrt{1-{\left(sin\;\varphi sin\alpha sin\omega +\cos\;\varphi \cos\alpha \right)}^{2}}$$

These expressions take into account that some Ga flux may impinge onto the NW sidewalls even at cosξ<0. Evaluation of the integral given by Equation (2) for 〈sinξ〉 shows that

(5) $$\langle \sin\xi \rangle \approx \sqrt{1-cos^{2}\varphi cos^{2}\alpha -1/2sin^{2}\varphi sin^{2}\alpha }$$

is a reasonable approximation in the entire range of angles α from 0 (corresponding to vertical NW) to 90° (corresponding to in-plane NW).

Figure 5b,c show the contour plots of the normalized collection areas $S_{eff}=\cos\xi$ for the top facet and $S_{eff}=\sin\xi$ for the NW sidewalls depending on the NW tilt angle α and the substrate rotation angle ω at a fixed Ga beam angle φ=40°, as in our MBE system. Without averaging over ω, the collection areas correspond to MBE growth with no substrate rotation. These plots were obtained from Equations (3) and (4) containing no free parameters. Quite naturally, no Ga atoms are received by the NW top facet at α> 40° for large enough ω, while the maximum collection on the top facet corresponds to α≅φ at ω around zero. The maximum collection on the NW sidewalls corresponds to in-plane NWs with α close to ±90°. Figure 5d shows the normalized contributions into the total axial growth rate originating from the top and side NW facets, averaged over the substrate rotation angle ω, versus the NW tilt angle α at a fixed φ of 40°. As expected, the top collection decreases and the sidewall collection increases as the NW tilt angle increases.

**Figure 6:**
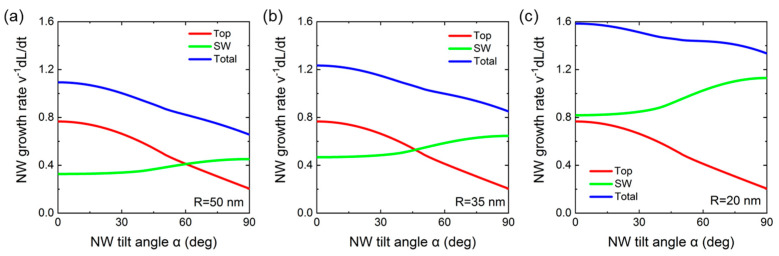
Total axial NW growth rates in the units of v, deconvoluted into the contributions of the top and sidewall (SW) facets versus the NW tilt angle. The curves are obtained from Equation (1) at a fixed Ga diffusion length $\lambda_{SW}$ = 40 nm and φ = 40° for different NW radii: (**a**) R= 50 nm, (**b**) R= 35 nm, and (**c**) R= 20 nm. In all cases, the total axial growth rate decreases with α. The decrease is steeper for larger NW radii.

**Figure 7:**
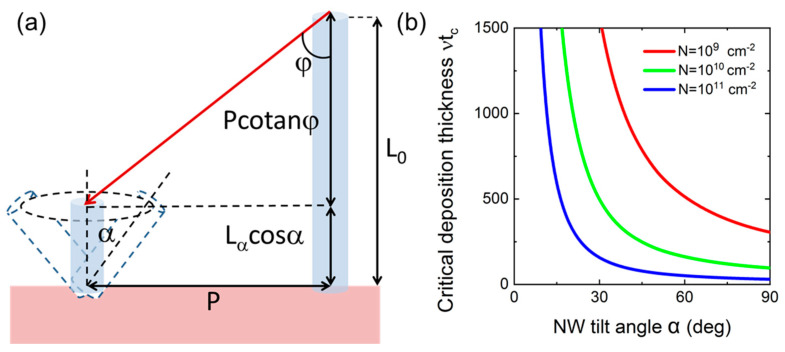
(**a**) Illustration of the geometrical shadowing effect on an NW with the tilt angle α, originating from the circle of neighboring vertical NWs. The rotating substrate does not influence the height of vertical NWs $L_{0}$, but reduces the effective height of inclined NW to $L_{\alpha }cos\alpha ,$ because for each rotation angle ω, the full length$\;L_{\alpha }$ is partly shadowed by one vertical NW in a circle. The average distance between the NWs equals P. From geometrical considerations, the full shadowing of a given NW occurs when its height plus Pcotanφ equals the length of vertical NWs. (**b**) Critical deposition thickness of GaN $vt_{c}$ as a function of the NW tilt angle at a fixed φ= 40° and three different NW densities given in the legend. The critical time $t_{c}$ at which all the NWs with the tilt angle α stop growing strongly decreases with α and N. Therefore, more inclined NWs stop growing faster, and finally only vertically aligned NWs survive in the whole NW ensemble.

Figure 6 shows the contributions to the NW axial growth rate originating from the direct impingement onto the top facet and surface diffusion of Ga adatoms from the NW sidewalls at a fixed $\lambda_{SW}=$40 nm and three different different NW radii R= 20 nm, 35 nm (corresponing to the average radius of GaN NWs in Figure 2), and 50 nm. The diffusion-induced axial growth rate increases and the direct impingement term decreases with the NW tilt angle, as in Figure 5d. The diffusion-induced contribution becomes greater for smaller NW radii. This leads to a steeper decrease in the total NW growth rate for larger NW radii. In all cases, the total axial growth rate decreases with the NW tilt angle, which is why more inclined NWs grow slower than less inclined NWs. At R= 35 nm, the axial growth rate of in-plane NWs decreases by approximately 30% with respect to the maximum growth rate of vertical NWs. The effective height of inclined NWs with a tilt angle α above the substrate surface, $H_{\alpha },$ is given by$\;H_{\alpha }=L_{\alpha }cos\alpha$ (see Figure 7a). Therefore, the longest vertically aligned NWs with $H_{0}=L_{0}$ will shadow the shorter inclined NWs in the course of MBE growth, leading to the geometrical selection of vertical NWs after a certain time. The shadowing effect should be enhanced in dense ensembles of NWs. 

To describe the effect of geometrical selection of vertically aligned NWs in MBE growth with substrate rotation, we consider the geometrical shadowing of a given NW by the neighboring NWs [44]. An NW with the tilt angle α gets fully shadowed by the longest vertical NWs when Ga atoms can no longer impinge onto its top surface and side facets. According to Figure 7a, this occurs when

(6) $$L_{\alpha }cos\alpha +Pcotan\varphi =L_{0}$$

where $P=1/\sqrt{cN}$ is the average distance between the NWs, N is their surface density, and c is a shape constant in the order of unity. Both$\;L_{\alpha }$ and $L_{0}$ are proportional to the actual NW growth time $t:\;L_{\alpha }=vf_{\alpha }t$ and $L_{0}=vf_{0}t$. Here,$\;f_{\alpha }$ is the α−dependent geometrical function shown in Figure 6 for R= 35 nm, which describes the axial growth suppression with increasing tilt angle α. Using these expressions, the critical NW growth time corresponding to the full shadowing of NWs with the tilt angle α, $t_{c}\left(\alpha \right)$, is obtained in the form

(7) $$t_{c}\left(\alpha \right)=\frac{1}{v\sqrt{cN}}\frac{cotan\varphi }{f_{0}-f_{\alpha }cos\alpha }$$

This simple expression shows that the critical time for the full shadowing of NWs (i) decreases with the Ga beam angle φ, (ii) decreases for higher NW density N, and (iii) decreases for larger NW tilt angles α. The latter property is due to (i) $f_{0}>f_{\alpha }$ corresponing to a slower axial growth rate of inclined NWs, and (ii) the presence of cosα in the denominator of Equation (7) describing the reduced height of inclined NWs above the substrate surface which decreases for more inclined NW and equals zero for planar NWs with α= 90°. 

Figure 7b shows the effective deposition thickness of GaN $vt_{c}\left(\alpha \right)$ as a function of the NW tilt angle α, obtained from Equation (7) at three different NW surface densities N of 10^9^ cm^−2^, 10^10^ cm^−2^ and 10^11^ cm^−2^ (corresponding to the highest NW density in Figure 2 and Figure 4d). It is seen that $vt_{c}\left(\alpha \right)$ gradually decreses with α and N. A larger suppression of more inclined GaN NWs at higher NW surface densities is confirmed by our experimental data shown in Figure 4. A more qualitative analysis of the effect of geometrical selection of vertically aligned GaN NWs in MBE growth as a function of the NW growth time and other technologically controlled parameters requires more studies and will be presented elsewhere.

## 4. Conclusions

To summarize, we have studied the morphology of GaN NWs grown by PAMBE on polycrystalline ZrN buffer layers. Our results lead to the following scenario of PAMBE growth of GaN NWs epitaxially linked to grains of the polycrystalline ZrN buffer. The NWs nucleate with random orientations induced by the metallic grains. The axial growth rate is highest for vertically aligned NWs, and decreases with increasing the NW tilt angle with respect to the substrate normal. At the beginning, the NWs continue to grow with random orientations as nucleated. At a certain stage, the effect of preferential growth induced by unidirectional supply of Ga in MBE starts to dominate. Fast-growing NWs with smaller tilt angles shadow the more tilted ones that grow slower. In this way, the NWs nearly perpendicular to the substrate surface are geometrically selected and survive in the entire NW ensemble. After a given growth time, the effect of geometrical selection of vertically aligned NWs is more pronounced for higher NW surface densities, which enabled us to obtain application-relevant dense ensembles of nearly vertical GaN NWs. Overall, we have demonstrated that the geometry of MBE growth helps to overcompensate for the random orientation of the initial GaN NWs induced by arbitrary arrangement of substrate grains. The practical implication of our finding is that for devices requiring a set of dense vertical NWs, an application of cheaper, polycrystalline buffer layers is sufficient. If, however, well-aligned sparse NWs are needed, a monocrystalline metallic substrate must be used, making the whole NW formation procedure much more complicated. Finally, we note that the effect of geometrical selection of vertically aligned NWs in the directional MBE technique is quite general, and should be present in different material systems where NWs nucleate with random orientations induced by the surface grains.

## Data Availability

The data presented in this study are available on request from the corresponding author.
